# Hypodermic Injections of Cold Water for Relief of Pain

**Published:** 1876-08

**Authors:** 


Hypodermic Injections of Cold Water for Relief of Pain.—There appeared in the February number of the *Medical News and Library* an extract from a communication in *L'Union Medicale*, by Dr. Lelut, on the relief of pain by hypodermic injections of cold water. This appeared to Dr. S. Henry Dessau not only a novel but such a ready method for the relief of pain, that he determined to give it a trial at the very first opportunity, and adds his testimony to the efficacy and promptness of the remedy by reporting, *New York Med. Journ.*, June, 1876,) seven cases in which it was most successfully used.—*Medical News and Library.*

